# Notes on the Augusta Meeting of the State Medical Association

**Published:** 1901-05

**Authors:** 


### NOTES ON THE AUGUSTA MEETING OF THE STATE MEDICAL ASSOCIATION.

THERE was an average attendance. The meetings were held in the city court room.

Dr. Benedict made a first-class presiding officer. He was well up on parliamentary law, and his rulings were made in a quiet, firm and courteous manner.

The program was arranged in a manner which gave considerable dissatisfaction—the eye, ear, nose and throat men having the best of it. There is always trouble in getting all the papers read. There should be formed a "steering committee" to arrange all complaints and fix a time for the reading of all papers which may not have been read in the position assigned.

The Association should absolutely refuse to extend any reader's time beyond the limit of twenty minutes.

Allowing a loss of half an hour of the time for the reading of a paper on oral hygiene, or dental details, was courteous, but a regrettable injustice to the members on the program. The reader was not a member, a dentist, and, as far as his paper could be heard, offered nothing new.

The barbecue was a quiet and pleasant affair, the strongest liquid served being coffee.

The remainder of the afternoon, after the barbecue, was lost. Perhaps the members expected to be unable to transact business. A night session was held to make up the time, but helped very little.

The report of the committee who audited the treasurer's accounts consumed a lot of time, but afforded some sensations and some amusement. There were two expert accountants' reports and things got mixed; so did some of the members who were not good at figures. One of the members tried to get in a motion and amendments, etc., until he got himself "balled up," and mighty near got the President in the same fix.

Dr. Baird will make an excellent and faithful president. If he is as good a presiding officer as the retiring president he will be good enough.

The drug and instrument exhibits were very few. Of our advertisers only Sharp & Dohme, The G. F. Harvey Co., represented by Mr. Wilson, and The Pheno-Bromate Co., Mr. Andrus, had exhibits. These were doing hard work and pushing their products in the right way.

Hotel accommodations were rather scarce for late arrivals. The Planters' was full up and the Bon Air, while having room enough, was a little too far away, though quite a number went there. The new Albion Hotel was not open.

We are told that Augusta doctors "dassent" belong to clubs or go into barrooms; a good place to live. Atlanta occupies a middle ground, while we are informed that Savannah goes to the other extreme. We hope to be able to testify more accurately about Savannah after next year's meeting.

